# Diagnostic yield and variant reassessment in the genes encoding Nav1.5 channel in Russian patients with Brugada syndrome

**DOI:** 10.3389/fphar.2022.984299

**Published:** 2022-08-24

**Authors:** Elena Zaklyazminskaya, Anna Shestak, Dmitry Podolyak, Vera Komoliatova, Leonid Makarov, Anna Novitskaya, Amiran Revishvili

**Affiliations:** ^1^ Petrovsky National Research Centre of Surgery, Moscow, Russia; ^2^ Bochkov Research Centre for Medical Genetics, Moscow, Russia; ^3^ Centre of Syncope and Cardiac Arrhythmias in Children and Adolescents, Moscow, Russia; ^4^ Sechenov First Medical State University, Moscow, Russia; ^5^ Vishnevsky Institute of Surgery, Moscow, Russia

**Keywords:** SCN5A, Nav1.5 channel, Brugada syndrome comorbidity, Brugada syndrome, cardiac channelopathy

## Abstract

Brugada syndrome (BrS) is an inherited cardiac arrhythmia characterized by ST-elevation, negative T-wave, and a high risk of sudden cardiac death (SCD) due to ventricular tachycardia. It is associated with mutations in over 20 genes but only *SCN5A* is recommended for routine genetic screening. This study was performed to estimate diagnostic yield and pathogenicity assessment of rare genetic variants in the genes encoding Nav1.5 channel in Russian patients with Brugada syndrome (BrS). Targeted genes panel sequencing of the five genes were screened using IonTorrent PGM with following Sanger confirmation. Detailed clinical evaluation of 75 unrelated BrS probands with a deep phenotyping of SCN5A (+) probands was performed. Twelve rare genetic variants (six missense, six truncating) were initially identified and classified as disease-causing. Reassessment of the clinical significance in the light of the current guidelines revealed: 2 Pathogenic (P) variants; 8 Likely Pathogenic (LP); two missense variants (p.G274S and p. S1778H) were re-classified later as a variant of uncertain significance (VUS). Unique VUS (p.Arg100Ser) was detected in the *SCN4B* gene. Lone Brugada-pattern was observed in 46% probands; 54% patients had concomitant arrhythmias. PR interval, the only electrocardiography parameter correlating with SCN5A-mutation, was longer (207 ± 24 ms) than normal in SCN5A (+) probands. SCD cases were registered in 31 families. Depression was the only recurring extra-cardiac complaint in SCN5A (+) probands; it was self-reported in five SCN5A (+) probands, and co-segregated with Brugada pattern in 2 families. After variants reassessment, the ratio of SCN5A (+) probands with Brugada syndrome accounts for 13% in Russian cohort.

## Introduction

Elevation of the ST segment in the right precordial leads in the absence of cardiac ischemia was noticed and described in 1953 as a rare variation of normal electrocardiograph (ECG) (Martini et al., 1989; Baranchuk, 2018). Forty years later, in 1992, P. and J. Brugada recognized this feature as a hallmark of a new inherited arrhythmic syndrome and complemented the description with pseudo-right bundle branch block (RBBB), T-wave inversion, and high risk of sudden cardiac death (SCD) due to polymorphic ventricular tachycardia (VT) (Brugada et al., 1998). Nowadays this disease named Brugada syndrome (BrS) counts as a cause of the 12% SCD events, and presumably underlies 20% of autopsy-negative cases (Juang and Huang, 2004). Its prevalence varies in different countries and ethnic groups with increasing gradient from Northwest to South East, with an intermediate rate between <0.5 per 1,000 individuals in United States and Western Europe and >3 per 1,000 individuals in Southeast Asia and Japan (Nademanee et al., 1997; Vutthikraivit et al., 2018). The incidence and prevalence of BrS in Russia are poorly understood. There was a single epidemiological study in the Central Volga (Samara Region) where ECG screening revealed 44 spontaneous Brugada-patterns in 47,000 inhabitants (Dupliakov et al., 2007). This corresponds to a prevalence of 0.9 per 1,000 individuals and it seems to be intermediate between Northern Europe and Asian populations.

The genetic heterogeneity of BrS is remarkable (Probst et al., 2009). By now mutations have been identified in two dozen other genes, except for *SCN5A*, encoding sodium, potassium, and calcium channels that are also known to be responsible for BrS. The mutation rate does not exceed 2–5% for most of them. Even screening of complete genes panel allows detection of pathogenic genetic variants in less than 50% of index cases. For routine screening of suspected BrS patients only the *SCN5A* gene is recommended without any ethnic specificity (Liu et al., 2010). This gene encodes the alpha subunit of the sodium channel Na_v_1.5 that plays a key role in the depolarization of cardiomyocytes. This channel also consists of four regulatory beta-subunits encoding by the *SCN1B-SCN4B* genes. A few publications describe mutations in these genes in BrS (Gottschalk et al., 2016; Dendramis et al., 2017; El-Battrawy et al., 2019).

Clinical manifestation of BrS also varies widely from syncope, ventricular and supraventricular arrhythmias, and cardiac arrest to asymptomatic longevity; therefore, risk assessment is still challenging. To date, implantable cardioverter-defibrillator (ICD) is considered the only proven method to reduce the risk of life-threatening events in these patients (Sacher et al., 2006; Benito et al., 2008). Some studies mention that extra-cardiac complications (i.e., gastroenterological complaints) may be a part of a more complex phenotype in patients with mutations in the *SCN5A* gene (Locke et al., 2006); but most published studies limit clinical description exclusively by arrhythmic presentation.

In this study, we present the results of molecular genetic screening of the genes encoding all subunits of the Na_v_1.5 channel (*SCN5A*, *SCN1B*, *SCN2B*, *SCN3B*, and *SCN4B*) in a pilot cohort of BrS probands of Russian origin. We also focused on deep phenotyping of the genotype-positive patients to better understand the phenotype-genotype correlation in Brugada Syndrome.

## Materials and methods

### Ethics declaration

The head of the local Ethics Committee of Russian Scientific Center of Surgery signed a permit on 27/09/2019 to perform this retrospective study on Brugada syndrome. This study was performed in accordance with the 1964 Helsinki declaration. Written informed consent was obtained from all individual participants included in the study. For patients under 18, consent was taken from a parent and/or legal guardian.

### Clinical investigation

Data obtained from each individual in the study included a personal and family medical history, general examination, 12-lead resting ECG, 24-h ECG Holter monitoring, transthoracic echocardiography (EchoCG), cardiac magnetic resonance imaging (MRI) with gadolinium enhancement, and pharmacological challenge test with class III anti-arrhythmic drugs (Sieira et al., 2015). The diagnosis of BrS was established based on current diagnostic criteria (Bayés de Luna et al., 2012).

### Genetic screening

Genetic screening of targeted genes panel encoding alpha- and beta-subunits of Nav1.5 (*SCN5A, SCN1B, SCN2B, SCN3B,* and *SCN4B*) was performed by semi-conductive sequencing using IonTorrent PGM. Confirmation of all variants detected by nest generation sequencing, resequencing of the low-coverage regions, and cascade familial screening were performed by bidirectional capillary Sanger sequencing. Sequencing data were analyzed using Torrent Suite (version 5.0.5), Ion Reporter annotation service (Thermo Scientific, Waltham, MA, United States), and the Integrative Genomic Viewer visualization tool. Analysis *in silico* of clinically relevant findings was performed using population databases (gnomAD, and 1,000 Genomes), missense variant effect predictors (SIFT, PolyPhen2, MutationTaster, etc.), splicing analysis tools (UMD HSF v3.1, NetGene2, SpliceAI), and data integrators (Varsome). Variants identified in 2009–2015 were assessed according to the traditional rules as “Mutation”, “Polymorphism” or “Rare variant”. Variants identified later were assessed based on American College of Medical Genetics and Genomics (ACMG) consensus recommendations (2015) (Richards et al., 2015). Variant reassessment found in BrS patients in 2009–2019 years was performed based on ACMG2015 guidelines and the latest refined recommendation (Richards et al., 2015; Walsh et al., 2021). Variants classified as “pathogenic”, “likely pathogenic”, and variants of unknown significance (Class III-V) were included into the final report for patients.

## Results and discussion

### Clinical polymorphism of Brugada syndrome in Russian probands

Seventy-five unrelated patients with Brugada syndrome (BrS) requested genetic counselling and DNA-diagnostics. Diagnosis of BrS was established in the 75 index patients based on current diagnostic criteria (2012, 2013) (Bayés de Luna et al., 2012; Priori et al., 2013). Spontaneous Brugada pattern type 1 was found in 29 out of 75 probands (38.6%). Their age ranged from 4 to 63 years at the time of initial diagnosis (30 ± 14 years). Male to female ratio was 6:1 (64 males and 11 females). Similar male gender predominance (3:1 in Europe and 9:1 in South Asia) was found in clinical observations worldwide (Nademanee et al., 1997). The follow-up was from 2 to 18 years but about 50% of patients fallen out from the follow up for different reasons.

Sporadic cases of BrS (single case in a family, no family history of SCD) were registered in 18 probands (24%). In 35% cases, familial data were incomplete or unavailable. A family history of SCD and/or Brugada pattern on ECG was reported by 31 probands (41%). Autopsy data from relatives died suddenly or their archive ECGs were only available in a minority of cases. For 10 adult probands and for the parents of a 5-year-old proband, information about SCD cases in the family became meaningful only in the context of genetic counseling. Fewer than half of the probands underwent systematic health screening within 1 year after the unexplained death of a relative. The relatives of the victims of SCD do not consider this a reason for clinical evaluation. A lack of information about the genetic background of SCD in society might be an important factor influencing the time of correct diagnosis and further compliance in cases of inherited arrhythmic syndromes.

Thirty-eight probands (51% of the whole cohort) including 3 children diagnosed before age 7 were asymptomatic and had no complaint. The reason for clinical and genetic evaluation in this subgroup was the occasional detection of the spontaneous Brugada pattern on resting ECG in otherwise healthy active people during professional, sport or preventive check-up, and pre-school examination.

**FIGURE 1:**
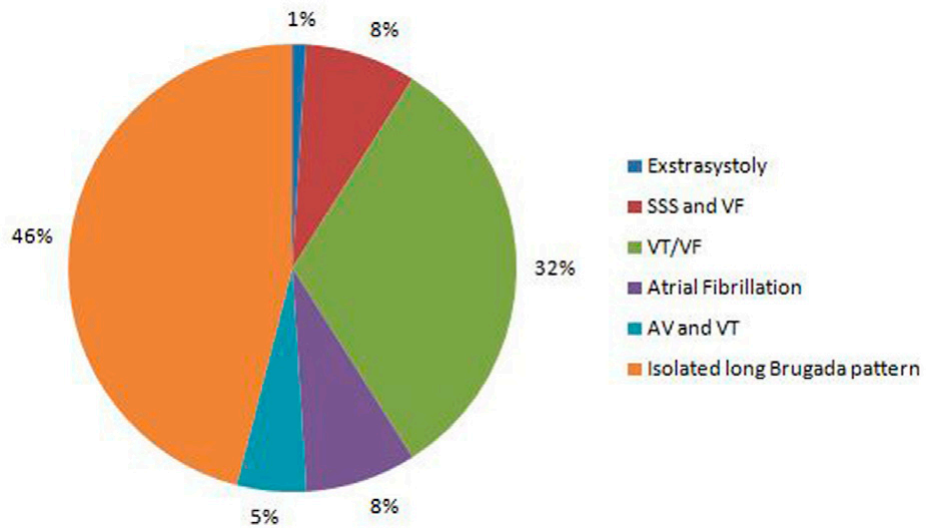
Spectrum of cardiac arrhythmias found in BrS patients.

Thirty-seven probands (49% of the whole group) underwent detailed clinical and genetic evaluation because of clinical symptoms. The most common complaints were syncope/pre-syncope (45%), palpitation (45%), dizziness (32%), and chest pain (30%) (Figure 1). Most individuals had more than one clinical symptom. Six probands (16% symptomatic patients, and 8% of the whole BrS cohort) survived cardiac arrest. Only three patients considered high-temperature conditions as a trigger for syncope (fever in one case and sauna in two cases), but many probands reported avoiding the sauna or other high-temperature conditions owing to “usual non-tolerance.” Five probands pointed out that moderate alcohol intake revealed chest pain, triggered syncope/cardiac arrest, and unmasked the Brugada pattern on ECG (none had reported alcohol addiction). This factor was previously described as a potentially underestimated trigger of BrS manifestation (Achaiah and Andrews, 2016).

Eight probands in symptomatic subgroup (11% of the whole BrS cohort) underwent at least one hospitalization in the intensive care department with a misdiagnosis of myocardial infarction and were discharged due to a lack of evidence for the ischemic origin of the phenotype and typical ECG dynamics. Transitory ST-segment and J-point elevation can mimic acute coronary episode, and differential diagnosis might be challenging (Abriel et al., 2000; Liu et al., 2010). It was shown that even expert cardiologists cannot always distinguish betwenn true Brugada syndrome and its phenocopies (Gottschalk et al., 2016). We assume that medical professionals, other than those with specific expertise, might be not sufficiently aware of BrS.

The Brugada pattern alone was observed in 46% probands. Most of patients (54%) had a wide spectrum of concomitant rhythm and conduction defects, including atrial fibrillation, premature ventricular contractions (PVC), non-sustained ventricular tachycardia (VT), ventricular fibrillation (VF), sick sinus syndrome (SSS), atrio-ventricular block (AVB) I-II degree, left bundle branch block (LBBB), and right bundle branch block (RBBB) (Figure 1).

Cardioverter-defibrillators were implanted in 8 probands (11%); 3 of them had appropriate shocks (1 male and 2 females). In one patient (NRF17, male, 38 y. o.) ICD was implanted in year 2011 because of spontaneous Brugada pattern and documented episode of non-sustained VT. After 6 years of episode-free follow-up and disappearance of the Brugada-pattern on ECG device, it was explanted by the agreement with patient. No QTc prolongation was ever registered. This proband is a carrier of heterozygous p. S1787N (rs199473316) variant initially described as a LQTS-causing mutation (Splawski et al., 2000). Cellular biophysical phenotype of this variant may vary significantly depending upon splice variant background and intracellular pH (Hu et al., 2015). Later it was shown that S1787N allele is associated with variable expressivity of BrS phenotype in *SCN5A* families, and can be considered as a risk factor (Wijeyeratne et al., 2020).

This observation enriches a list of pathogenic mutations affecting amino acid sequence of the Na_v_1.5 alpha subunit that may result in both, LQTS and Brugada syndrome (in-frame deletion ∆KPQ is the most known example) (Saber et al., 2015; Abdelsayed et al., 2017). Currently (2019) this rare genetic variant was reclassified as a variant of unknown significance (VUS) (Class III) (Glazer et al., 2020). The patient was invited for genetic counseling regarding variant reclassification.

Structural heart disease was found in six probands (8%). Three probands (2 females, one male) additionally met the diagnostic criteria (2010) for arrhythmogenic right ventricular cardiomyopathy (ARVC) (Marcus et al., 2010). Two out of 3 underwent radiofrequency ablation of the arrhythmogenic focus in the right ventricular outflow tract (RVOT). One male patient had dilated cardiomyopathy (DCM) with left ventricular enlargement and decreased ejection fraction up to 40%. Asymptomatic non-obstructive hypertrophic cardiomyopathy (HCM) was revealed by EchoCG and cardiac MRI in 2 male probands. One *SCN5A* (+) male proband was first diagnosed with unexplained cardiac hypertrophy in 2011 (38 y. o.) when IVS reached 14 mm (38 y. o.). During last 9 years of follow-up he remains asymptomatic but non-obstructive HCM is slowly progressing. Now IVS revealed 23 mm, LV posterior wall 13 mm, and RV wall 11 mm. Another male proband, SCN5A (-), had interventricular septum (IVS) up to 15 mm and no obstruction.

There are publications discussing phenotypic overlap between BrS and ARVC, and possible inter-relationship caused by a cross talk between desmosomal and sodium channel proteins (Corrado et al., 2016). Dilated cardiomyopathy as a part of phenotype in patients with both loss-of-function and gain-of-function mutations in the *SCN5A* gene, is also widely discussed (Wilde and Amin, 2018). But cardiac hypertrophy seems to be exclusively rare phenomenon in patients with channelopathies. We performed search through the current literature and found two clinical case reports of HCM patients: one with rare variant (p.Asp872Asn, VUS) in the *SCN5A* gene (no arrhythmic phenotype mentioned) (Cronin et al., 2018), and second patient with Brugada pattern on ECG and two rare variants in the *MYH7* and *MyBPC3* genes (Farnè et al., 2021). Whether BrS and HCM may have a common pathogenic mechanism remain unsolved. Mild cardiac hypertrophy might be an overlooked feature in patients with threatening diagnosis such as Brugada syndrome in these two observations.

We cannot exclude an occasional combination of two different genetic conditions (BrS and HCM; BrS and ARVC; BrS and DCM) in patients from our BrS cohort. It was known that multilocus variations can be found in more than 30% of patients with unusual phenotype (so called “phenotypic expantion”) (Karaca et al., 2018).

### Genetic testing results and pathogenicity reassessment

No mutation was found in *SCN1B-4B*, suggesting that these mutations are very rare in Russian patients. A unique heterozygous variant, p. R100S, in *SCN4B* was found in a 30-year-old proband of mid-Asian origin with the spontaneous Brugada type-1 pattern and 2 pre-syncopal episodes.

All *in silico* predictive tools (PolyPhen 2.0, SIFT, and MutationTaster) classify these variants as “disease causing” or “probably damaging.” In the absence of familial and functional data, further study is needed to evaluate the possible role of these variants in BrS pathogenesis.

Twelve rare variants (6 truncating and six missense) were found in *SCN5A* in 12 male probands (16%), and 11 out of 12 rare variants were considered related to the phenotype at the moment of detection (2009–2015) (Table 1). Eight variants were reported to patients as “mutations”, 3 variants (detected after 2015) were reported as Likely Pathogenic (LP), and one variant was considered as “Rare variant” (corresponds to VUS in current terminology).

**TABLE 1:** Summary of *SCN5A* rare variants identified with Brugada syndrome. Classification of variant pathogenicity is according to the American College of Medical Genetics (ACMG) joint consensus standards and guidelines for the interpretation of sequence variants. All genetic variants were referred for a single transcript NM_198,056.2.

Code of the family	Coding sequence	Protein sequence	References sequence	GnomAD MAF	Initial assessment at the time of detection (before guidelines)	Reassessment based on guidelines (criteria chosen provided) (18, 18-1)
NRF 15	c.260A>G	p.Y87C	-	-	“Rare variant”	LP (PS3, PM2,PP3)
NRF 129	c.820G>A	p.G274S	rs794728852	0.00003184	LP	VUS (PM1, PM2, PP3)
NRF 191	c.999-1G>A	Splice site disruption	-	-	LP	LP (PVS1, PM2)
NRF 89	c.1233del	Frameshift deletion	-	-	“Mutation”	LP (PVS1, PM2)
BrS 15	c.1657G>T	p.E553*	rs1207394743	0.00003186	“Mutation”	P (PVS1, PM2, PP1)
BrS 87	c.2542_2544del	p.I848del	-	-	“Mutation”	LP (PM1,PM2,PM4)
NRF 10	c.2678G>A	p.R893H	rs199473172	0.000003986	“Mutation”	P (PM1,PM2,PP3,PP5)
BrS 101	c.4299 + 1G>A	Splice site disruption	-	-	“Mutation”	LP (PVS1, PM2)
NRF 88	c.4642G>A	p.E1548K	rs199473271	0.00003185	“Mutation”	LP (PM1,PM2,PP3,PP5)
NRF 179	c.4714 + 2C>T	Splice site disruption	-	-	LP	LP (PVS1,PM2)
NRF 85	c.4720G>T	p.E1574*	-	-	“Mutation”	LP (PVS1, PM2)
NRF 17	c.5360G>A	p.S1787N	rs199473316	0.0008290	“Mutation”	VUS (PS4,PP3)

LP, likely pathogenic; P, pathogenic; VUS, variant of unknown significance.

We reassessed all 12 rare variants in this cohort using ACMG (2015) criteria (Richards et al., 2015), Enhancing rare variants interpretation for inherited arrhythmias recommendations (Walsh et al., 2021), and additional published data available. All six truncating genetic variants (“mutations”) were reclassified as Pathogenic (P) (p.E533*) or Likely Pathogenic (LP) (c.999-1G>A, c.1233del, c.4299 + 1G>A, c.4714 + 2C>T, p. E1574*) (Table 1). Three out of six missense variants were reclassified based on ACMG criteria (2015, 2019). One missense variant, p. Y87C, was reclassified from “Rare variant” (=VUS, Class III) to Likely Pathogenic (P, Class V) because of the experimental evidence of deleterious effect on calmodulin binding with N-terminal domain of the Nav1.5 (Wang et al., 2020). Two missense variants, p. G274S and p. S1787N, were re-classified from “Mutations” (Class V/IV) to VUS (Variant of unknown significance, Class III) due to conflicting publications. All probands carrying these variants were invited for genetic counseling.

No mutation was found in a female proband; female gender of the proband is likely a negative predictive factor for the presence of *SCN5A* mutations.

### Genotype–phenotype correlations

Clinical characteristics of *SCN5A* (+) probands are summarized in Table 1. All truncating mutations (nonsense, frame-shift deletion, and splicing variants) in the *SCN5A* gene were of familial origin. Spontaneous Brugada pattern type 1 on the ECG in V_1_-V_2_ or V_1_’-V_2_’ was registered in all male carriers, including a young asymptomatic 4-year-old boy. Cases of SCD in families were reported for all probands harboring truncating mutations. The age at the time of the SCD or life-threatening VT in these families was 37–52 years. Four female family members carrying PTC mutations had Brugada pattern type 1 induced by pharmacological challenge test. Cascade familial screening revealed 12 additional family members (8 males, four females) carrying truncating variants, and all males had spontaneous Brugada pattern on ECG.

No significant differences in the cardiac manifestation of Brugada syndrome, such as the rate of syncope, SCD cases, age at manifestation, heart rate, QTc, spectrum of ventricular and supra-ventricular arrhythmias, and ICD implantation were found between genotype-positive and genotype-negative probands. The only parameter that differed significantly between these groups was the PR interval (207 ± 24 ms in *SCN5A* (+) probands vs. 172 ± 18 ms in *SCN5A*(−)probands, *p* < 0.01). These data are in accordance with the finding that in patients with BrS, a widening of the PQ interval (>210 ms) is predictive of the presence of *SCN5A* mutations (Smits et al., 2002).

**FIGURE 2:**
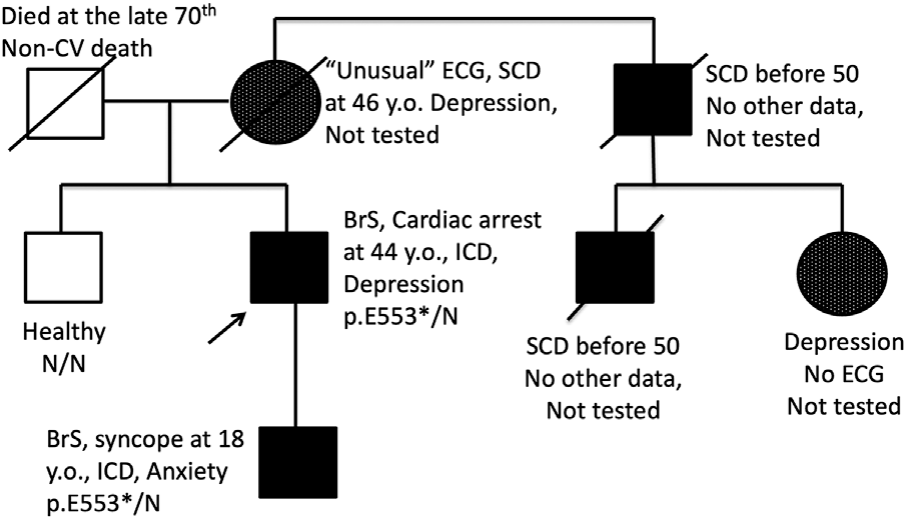
Pedigree of the family BrS15 with co-segregation of BrS pattern on ECG and endogenous depression. Proband and his son are carriers of heterozygous nonsense p. E553* mutation in the *SCN5A* gene. SCD, Sudden cardiac death; ↑, proband; □, male; ○, female.

Most of genes causing cardiac channelopathies have a wide expression profile and function in multiple tissue types. Thereby, extra-cardiac involvement is not unusual in cardiac channelopathies. We performed a survey of extra-cardiac complaints in patients with BrS. An increased prevalence of gut motility problems in *SCN5A* mutation carriers has been observed (Locke et al., 2006) but this symptom was not mentioned in the survey by any patient. No cases of proven epilepsy were found. Five probands (7%) self-reported at least one episode of depression requiring medication and/or hospitalization, and all these patients belonged to the *SCN5A* (+) group (Table 2). In family #BrS15, the recurrent depression co-segregated with BrS in 3 generations and the p.553* heterozygous mutation (Figure 2). In the family #NRF15 the co-inheritence of p. Y87C variant, Brugada Syndrome, and recurrent depression was found in 2 generation) (Wang et al., 2020).

**TABLE 2:** Clinical characteristics of *SCN5A* (+) probands (all males) carrying heterozygous mutations in the *SCN5A* gene.

Code of the family	Genetic variant	Age*, years	Brugada pattern, type 1	Syncope	ICD	CMP	BrS SCD in family	Extra-cardiac complaints
NRF 15	p.Y87C	23	spontaneus	-	-	-	BrS	Recurrent depression
NRF 129	p.G274S	37	induced	-	-	-	-	no data
NRF 191	c.999-1G>A	4	spontaneus	+	-	-	SCD	no data
NRF 89	c.1233del	5	spontaneus	+	+	-	BrS	Mood disorders
BrS 15	p.E553*	38	spontaneus	-	+	-	BrS, SCD	Recurrent depression
BrS 87	p.I848del	36	spontaneus	-	+	-	-	no data
NRF 10	p.R893H	27	spontaneus	+	+	-	SCD	Recurrent depression, panic attacks
BrS 101	c.4299 + 1G>A	38	spontaneus	+	+	-	-	Recurrent depression
NRF 88	p.E1548K	6	spontaneus	-	-	-	BrS	no
NRF179	c.4714 + 2C>T	28	spontaneus	-	-	-	-	no data
NRF85	p.E1574*	22	induced	-	+	-	-	no data
NRF17	p.S1787N	38	induced	-	+**	HCM	-	no

*Age at the first consultation.

**ICD, was implanted, and then it was explanted after 6 years of event-free period.

CMP, cardiomyopathy; HCM, hypertrophic cardiomyopathy; SCD, sudden cardiac death.

Na_v_1.5 channels encoded by *SCN5A* are over-expressed in some non-excitable and excitable cells in the central nervous system and may contribute to diverse cellular functions (Catterall, 2014). We speculate that emotional disorders might be more common in *SCN5A*-related BrS than previously thought, but this observation requires confirmation in a larger study. The clinical cases of BrS reveled in patients with depression were published since 2014 (Chen and Sangha, 2014). This might have implications for the treatment of depression because a number of psychotropic agents are included in the “to avoid” and “to preferably avoid” lists for patients with BrS (Postema et al., 2009) and should be taken with caution.

## Conclusion

After the rare variant reassessment, the prevalence of *SCN5A*-related BrS in Russian patients was 13%, consistent with the prevalence of *SCN5A*-associated BrS worldwide. Carriers of *SCN5A* mutations have a relatively longer PR interval. Female gender of proband is a negative predictive factor for mutations affecting Na_v_1.5 sodium channels. Genetic forms related to the beta-subunits are extremely rare in Russia, if any. Structural myocardial disorders were found in 8% of Russian BrS index cases. Further study is needed to evaluate if endogenous depression was an accidental finding of this group, or it really expands the clinical spectrum of *SCN5A*-related phenotype.

Risk stratification schemes and treatment strategies for patients with BrS are not currently based on genotypes but this could be a focus of future research. Preventive and therapeutic strategies will also benefit from increased knowledge of causal mutations and their consequences (Abriel et al., 2000). Current guidelines can be considered precursors to the development of further guidelines for genetic testing in cardiology and for a new approach for the integration of genetics and technology into clinical practice and personalized medicine.

## Data Availability

The original contributions presented in the study are included in the article/supplementary material, further inquiries can be directed to the corresponding author. The data presented are deposited in the repository ClinVar (https://www.ncbi.nlm.nih.gov/clinvar/). Accession numbers for registered variants VCV000691966.6, VCV000201445.6, VCV001348900.1, VCV000067749.6, VCV000067913.1, VCV000067988.32, and VCV000691965.2.
